# Genomic Insights into the Distribution of Peptidases and Proteolytic Capacity among *Prevotella* and *Paraprevotella* Species

**DOI:** 10.1128/spectrum.02185-21

**Published:** 2022-04-04

**Authors:** Amlan Kumar Patra, Zhongtang Yu

**Affiliations:** a Department of Animal Nutrition, West Bengal University of Animal and Fishery Sciences, Belgachia, Kolkata, India; b Department of Animal Sciences, Ohio State University, Columbus, Ohio, USA; University of Illinois at Urbana Champaign

**Keywords:** *Prevotella*, protease, protein degradation, ruminants, secretory peptidase, phylogenetic relationship, genome analysis, peptidases

## Abstract

Bacterial peptidases play important roles in health and nutrient digestion in both humans and animals, especially ruminant animals. In this study, we examined and compared the various peptidases (both total and secretory) among species of Prevotella (44 in total) and *Paraprevotella* (2) revealed in their sequenced genomes that were archived in the MEROPS database. The phylogenetic relationships were also compared among the species based on 16S rRNA gene sequences and the occurrence of peptidases. A rich repertoire of peptidases was found that represents six catalytic types of peptidases (aspartic, cysteine, glutamic, metallo, mixed, and serine), together with some with unknown catalytic mechanisms, and 78 families. Metallopeptidases were the most predominant, followed by serine and cysteine peptidases. Considerable variations in peptidase occurrence and distribution were noted among the species and the different families of peptidases. A total of 48 different families of secretory peptidases were found in the genomes of the Prevotella and Paraprevotella species. Secretory peptidases in the families of S41 and M13 were ubiquitous, and S9, M16, C1, S13, and C69 were found in more than 95% of the species. Multivariate analysis of the peptidases indicated that species were mostly clustered except for a few species. Analysis using a bipartite association network showed that the majority of peptidase families were shared among the species. The relatedness of peptidase distributions among the species did not significantly correlate with their phylogenetic relationship based on the 16S rRNA genes. The genomic overview on the peptidases of Prevotella and Paraprevotella species provided new insights into their potential capacity to degrade proteins.

**IMPORTANCE** Species of Prevotella are prevalent and predominant bacteria residing in animals and humans, and their proteolytic capacity and activity play important roles in nutrient utilization in animals (especially ruminants) and some anaerobic infections of the intestinal, respiratory, and urinary tracts in humans. This study reveals the large repertoire and wide distribution of metallo, serine, and cysteine peptidases, especially secretory peptidases, among the Prevotella species. The information presented here could aid in the identification of the Prevotella species and the peptidases to target to decrease the excessive protein degradation in the rumen and improve dietary nitrogen utilization by ruminant animals.

## INTRODUCTION

Bacterial degradation of both dietary and host-derived protein includes proteolysis, by which protein is depolymerized into smaller polypeptides or amino acids (AAs) and further catabolism of the proteolytic products. Bacterial protein degradation in the gastrointestinal (GI) tract is an important process in nutrient utilization and some diseases in humans and animals (1). In humans and nonruminant animals, bacterial degradation of dietary and host-derived protein occurs primarily in the large intestines, where most of the gut microbes reside (2), producing oligopeptides, AAs, and short-chain fatty acids (SCFAs), including branched-chain fatty acids (BCFA). Both AAs and SCFAs can be absorbed by enterocytes as nutrients, even though only a small amount of AAs can be absorbed by the large intestine (1). However, many of the oligopeptides and AAs are fermented by GI bacteria producing SCFAs and several toxic metabolites, including phenols, indoles, ammonia, sulfide, and amines, which are genotoxic and can increase the risk of colorectal cancer (3, 4). Proteolytic degradation mediated by bacterial peptidases is also involved in some other diseases (5). For example, proteolytic degradation of the extracellular matrix of the GI tract by bacteria is implicated in inflammatory bowel diseases (IBDs) in humans (5–7). Bacterial proteolytic degradation of host protein is implicated in periodontitis (8) and urinary tract infections (9). In ruminants, bacterial proteolytic degradation of dietary protein in the rumen plays a central role in converting dietary protein to microbial protein, which represents 60 to 85% of the AAs reaching the animal’s small intestine (10). Furthermore, microbial protein provides balanced AA profiles for milk and meat production (11–13). Therefore, bacterial proteolytic degradation of dietary protein is an essential process for microbial protein synthesis in the rumen. However, a significant portion of the proteolytic products, both oligopeptides and AAs, is fermented to SCFAs and ammonia or decarboxylated to amines due to the excessive degradation of dietary and microbial proteins (14). Although SCFAs are used as nutrients, much of the ammonia is converted to urea and excreted by the animals (15, 16), lowering nitrogen utilization efficiency (only about 30% in dairy cows). Furthermore, amines cannot be used by animals or humans, and they are also toxic. Species of Prevotella play an important role in protein degradation, especially in the rumen (17).

Prevotella species are anaerobic Gram-negative bacteria, and they inhabit the oral cavity, GI tract, and urinary tract of animals and humans (18–22). Prevotella is particularly predominant in the GI microbiome of humans with a Prevotella enterotype (23) and the rumen microbiome (21). Species of Prevotella are commensal, but several Prevotella species are implicated in some anaerobic infections of the GI tract, respiratory tract, and urinary tract (19, 22). For example, Prevotella dentalis, Prevotella melaninogenica, Prevotella denticola, Prevotella enoeca, Prevotella fusca, Prevotella intermedia, and Prevotella sp. oral taxon 299 are involved in periodontal diseases, such as gingivitis and periodontitis in the oral cavity of humans (20). In the rumen of ruminants, species of Prevotella are among the most predominant and proteolytic isolates (24, 25), and most of them are classified as Prevotella ruminicola, followed by Prevotella brevis, Prevotella bryantii, and Prevotella albensis. Based on quantitative PCR (qPCR) analysis, the genus Prevotella can account for 20 to 60% of the bacterial abundance and represents a substantial functional diversity of the rumen microbiome (18, 21, 26). However, P. ruminicola and P. bryantii represent only 2 to 4.4% of the total bacterial rRNA gene copies (18, 21), while P. brevis and P. albensis have a negligible abundance (21). Many Prevotella species evidently remain to be cultured and characterized. Indeed, most (88%) of the sequenced 16S rRNA gene clones prepared from rumen samples shared less than 97% sequence similarity with a known ruminal Prevotella species (18). In a phylogenetic census, there were 673 operational taxonomic units (OTUs, defined at 97% sequence similarity), but only 20 of them contained sequences from cultured/characterized Prevotella strains (27). Known rumen species of Prevotella produce various extracellular digestive enzymes, including amylases, xylanases, peptidases, and deaminase (28, 29), allowing them to be nutritionally versatile and grow on small peptides, many AAs, and sugars (21). Therefore, Prevotella species play significant roles in the digestion and fermentation of nutrients, especially dietary protein and noncellulosic polysaccharides (18, 21, 25). Peptidases are essential for the proteolytic activities of Prevotella species (30).

Peptidases (or proteases or proteinases) are hydrolytic enzymes ubiquitous among microbes (31–34). Peptidases are classified into different catalytic types based on the catalytic residues: aspartic peptidases (A), cysteine peptidases (C), glutamic peptidases (G), metallopeptidases (M), asparagine peptidases (N), mixed peptidases (P), serine peptidases (S), threonine peptidases (T), and unknown peptidases (U). Each catalytic type of peptidases is classified into different clans, each of which has arisen from a single evolutionary origin of peptidases (35). Each catalytic type is then classified into one or more families based on AA sequence homology. Chemical characterization of individual peptidases from individual bacteria is time-consuming and laborious. The genome sequences of bacteria provide an opportunity to examine the catalytic types and families, distribution, and abundance of peptidases that can be produced by individual bacteria. In the study presented here, we analyzed and compared the catalytic types and distribution of the peptidases identified in the sequenced genomes of Prevotella species. We also compared the phylogenetic relationships among individual Prevotella bacteria using both peptidase sequences and 16S rRNA gene sequences. Species of Paraprevotella, which is a genus taxonomically established in 2009, are phylogenetically and phenotypically most similar to Prevotella species (36), and they were also included.

## RESULTS

### Abundance and distribution of different catalytic types and families of peptidases.

**FIG 1:**
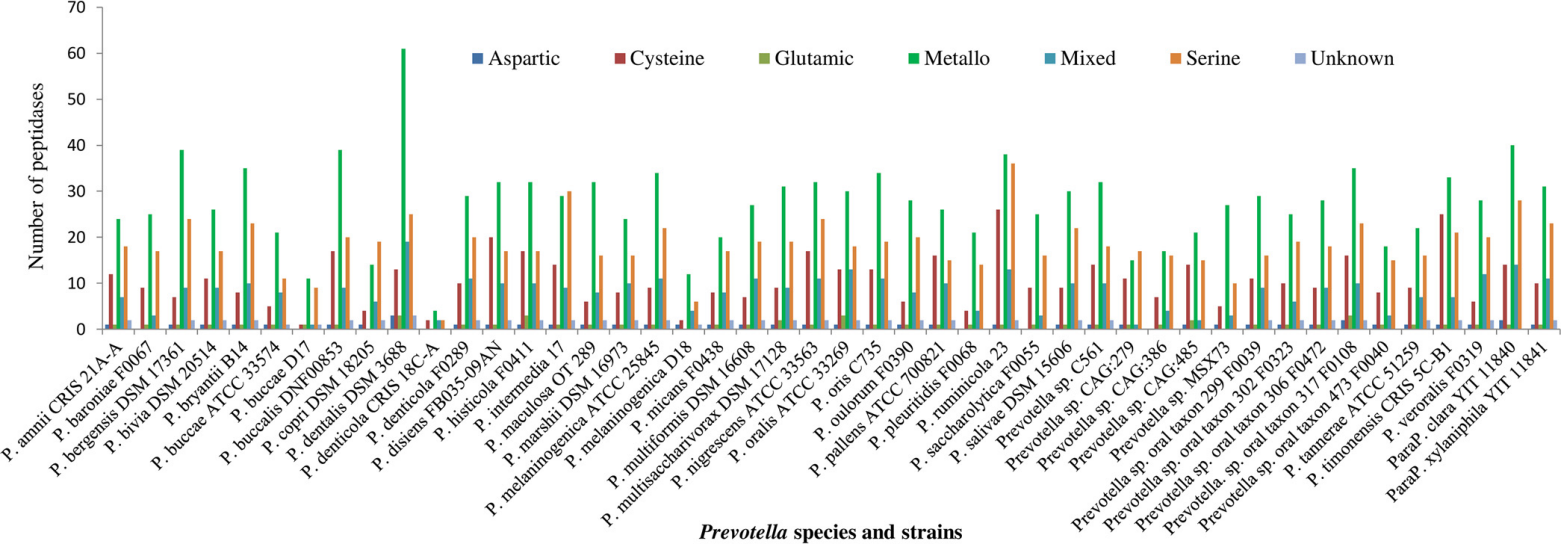
Catalytic types of peptidases found in the genomes of Prevotella (*P*.) and Paraprevotella (*ParaP.*) species and strains. Metallo, metallopeptidase.

The MEROPS database (https://www.ebi.ac.uk/merops/) is a specialty peptidase database of manually curated proteolytic enzymes (35). It has archived peptidases of 34 strains of Prevotella within 31 known species, 10 strains that have not been assigned to any known species, and 2 strains of 2 *Paraprevotlla* species (Fig. 1). A total of 3,131 peptidases and 550 nonpeptidase homologs were found in the genomes of the 44 strains of Prevotella and two strains of Paraprevotella. The nonpeptidase homologs were not further analyzed. All the known catalytic types of peptidases, except asparagine and threonine peptidase, were found in the sequenced genomes of the Prevotella and Paraprevotella species (Fig. S1). On average, each genome had (± standard deviation) 68.1 (±22.1) peptidases. The number of peptidases per genome varied considerably among the species, with the largest in P. dentalis DSM 3688 (*n* = 127) followed by P. ruminicola 23 (*n* = 117), and the smallest were in P. denticola CRIS 18C-A (*n* = 10) and P. buccae D17 (*n* = 24) (Fig. 1). The catalytic type of metallopeptidase was the most predominant in all the species except for P. intermedia 17 and Prevotella sp. CAG:279, in which serine peptidase was the most predominant (*n* = 29 versus 30 and *n* = 15 versus 17, respectively). Serine peptidase was the second most predominant in all the species except for Prevotella disiens FB035-09AN, Prevotella pallens ATCC 700821, and Prevotella timonensis CRIS 5C-B1, in which cysteine peptidase was the second most predominant. Aspartic peptidase and glutamic peptidase were the least prevalent and predominant (ranging from 0 to 3 per genome). The distribution of the catalytic types of peptidases also varied between some strains of the same species, such as P. buccae D17 versus P. buccae ATCC 33574 and P. denticola CRIS 18C-A versus P. denticola F0289 (Fig. 1).

A total of 78 distinct families of peptidases were found in the sequenced genomes of the Prevotella and Paraprevotella species, with 34 families being found in more than 50% of the species/strains (Table 1) and the remaining 44 being found in less than 50% of the species/strains (Table S1). Among these families, S9 (serine peptidase), M20 and M16 (metallopeptidase), and C10 and C26 (cysteine peptidase) were the most predominant families, with each representing more than 5% of the total peptidases found in all the species/strains. The families M16, S41, and C1 were the most prevalent in all the species (100%), and S9, M3, M13, M20, S13, and S49 were found in more than 95% of the species/strains. The families S13 and S49 were at low abundance, but they were found in 95.7% of the species/strains. Several families were found only in some of the species/strains, including 36 peptidase families that were found in less than 20% of the species/strains. The number of peptidases in a family varied considerably among the genomes, ranging from 0 to 17, with the largest count in the family C10 (*n* = 17) in P. timonensis CRIS 5C-B1 followed by S9 (*n* =16) in P. ruminicola 23, M20 (*n* = 15) in P. dentalis DSM 3688, S8 (*n* = 14) in P. intermedia 17, C26 (*n* = 12) in P. dentalis DSM 3688, and C10 (*n* = 12) in P. disiens FB035-09AN (Table S2).

**TABLE 1:** Different peptidase families present in *Prevotella* and *Paraprevotella* species/strains^*a*^

Peptidase family	Total no.	Percentage of total peptidases (%)	Total carrying species/strains	Percentage of total carrying species/strain (%)
M16	186	5.94	46	100
S41	139	4.44	46	100
C1	122	3.90	46	100
S9	219	6.99	45	97.8
M3	77	2.46	45	97.8
M13	74	2.36	45	97.8
M20	196	6.26	44	95.7
S13	57	1.82	44	95.7
S49	48	1.53	44	95.7
C69	65	2.08	43	93.5
S16	64	2.04	43	93.5
C25	47	1.50	43	93.5
G5	51	1.63	42	91.3
M49	45	1.44	42	91.3
M41	43	1.37	42	91.3
S8	118	3.77	41	89.1
M6	102	3.26	41	89.1
S1	50	1.60	41	89.1
S54	47	1.50	41	89.1
S26	46	1.47	41	89.1
A8	43	1.37	41	89.1
C10	187	5.97	40	87.0
M19	44	1.41	40	87.0
S46	41	1.31	40	87.0
C26	179	5.72	39	84.8
S14	47	1.50	39	84.8
M50	39	1.25	39	84.8
M24	129	4.12	37	80.4
M23	90	2.87	37	80.4
U32	70	2.24	36	78.3
M48	51	1.63	34	73.9
M15	33	1.05	31	67.4
C39	72	2.30	26	56.5
M93	38	1.21	25	54.3

a The peptidase families (of 78 in total) present in more than 50% of the bacterial species/strains are shown here.

### Abundance and distribution of different types of secretory peptidases.

**FIG 2:**
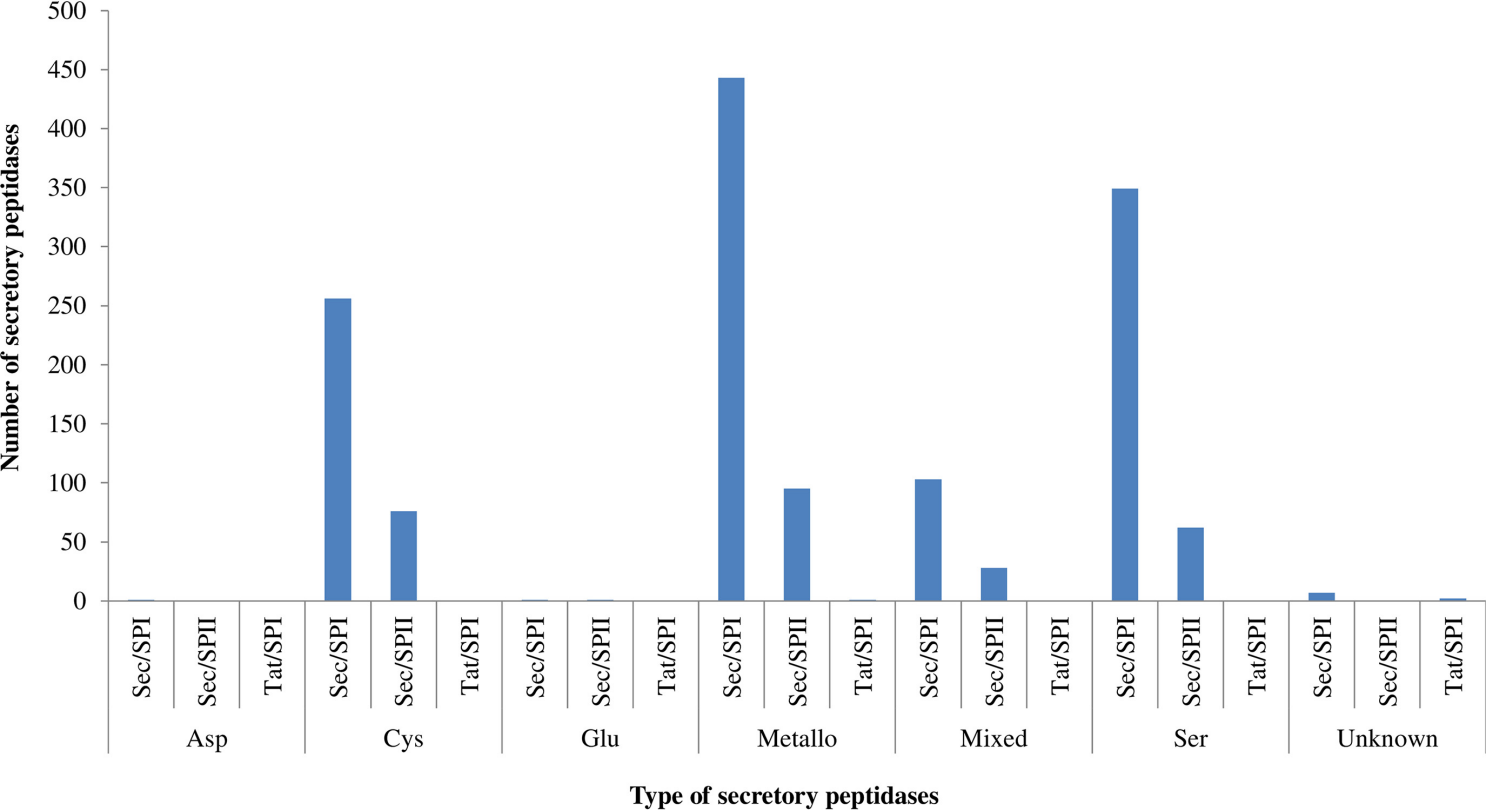
Secretory types of peptidases of different catalytic types found in the genomes of Prevotella and Paraprevotella species/strains. Asp, aspartate; Cys, cysteine; Glu, glutamate; Ser, serine; Sec/SPI, peptidases Sec/SPI, secretory peptidases with a signal peptide transported by secretion translocation and cleaved by signal peptidase I; Sec/SPII, secretory peptidases with a lipoprotein signal peptide transported by secretion translocation and cleaved by signal peptidase II; Tat/SPI, secretory peptidases with a Tat signal peptide transported by twin-arginine translocation and cleaved by signal peptidase I.

A total of 1,425 secretory peptidases, which represented 45.5% of the total peptidases, were found in the sequenced genomes of Prevotella and Paraprevotella species/strains. On average (± standard error), 33.1 (± 7.39) secretory peptidases were found per genome. Similar to the occurrence of total peptidases, secretory peptidases were mostly metallo (37.8%), serine (28.8%), and cysteine (23.3%) peptidase, the three of which together represented 90% of the total secretory peptidases (Fig. 2). Glutamic peptidase (0.07%) and aspartic peptidase (0.14%) were the least predominant. Among the secretory peptidases, the Sec/SPI secretory type was the most predominant (81.3%), followed by lipoprotein signal peptides transported by secretion translocation and cleaved by signal peptidase II (Sec/SPII) (18.4%), whereas the Tat signal peptides transported by twin-arginine translocation and cleaved by signal peptidase I (Tat/SPI) secretory type was sparse among the Prevotella and Paraprevotella species/strains (Fig. 2).

The secretory peptidases represented a total of 48 different families, including 18 prevalent with each having a prevalence of more than 50% (Table 2) and 30 families with each having a prevalence of less than 50% (Table S3). The secretory peptidases in the families S41 and M13 were ubiquitous (present in all species), and S9, M16, C1, S13, and C69 were found in more than 95% of the species/strain (Table 2). Those in families S9 (11.5%), C10 (10.2%), and M16 (9.26%) were most predominant, while many families were minor (less than 0.5% for 24 families combined). The number of secretory peptidases in a family varied among the species/strains, ranging from 0 to 12 (Table S4). Among all the species, the top 5 peptidase family with highest counts was the C10 family, which was present in P. timonensis CRIS 5C-B1 (*n* = 12), P. disiens FB035-09AN (*n* = 9), Prevotella histicola F0411 (*n* = 9), Prevotella buccalis DNF00853 (*n* = 8), and P. ruminicola 23 (*n* = 8) (Table S4).

**TABLE 2:** Different secretory peptidase families found in Prevotella and Paraprevotella species/strains^*a*^

Peptidase family	Total no.	Percentage of total secretory peptidases (%)	Total carrying species/strains	Percentage of total carrying species/strains (%)
S41	84	5.89	43	100.0
M13	65	4.56	43	100.0
S9	164	11.51	42	97.7
M16	132	9.26	42	97.7
C1	107	7.51	42	97.7
S13	52	3.65	41	95.3
C69	43	3.02	41	95.3
M6	88	6.18	40	93.0
C10	145	10.18	39	90.7
S1	45	3.16	39	90.7
S8	83	5.82	36	83.7
S46	36	2.53	36	83.7
M23	75	5.26	35	81.4
C25	38	2.67	35	81.4
M15	27	1.89	27	62.8
M93	40	2.81	26	60.5
M48	30	2.11	25	58.1
M3	26	1.82	24	55.8

a The secretory peptidase families (of 48 in total) present in more than 50% of the bacterial species/strains are shown here.

**FIG 3:**
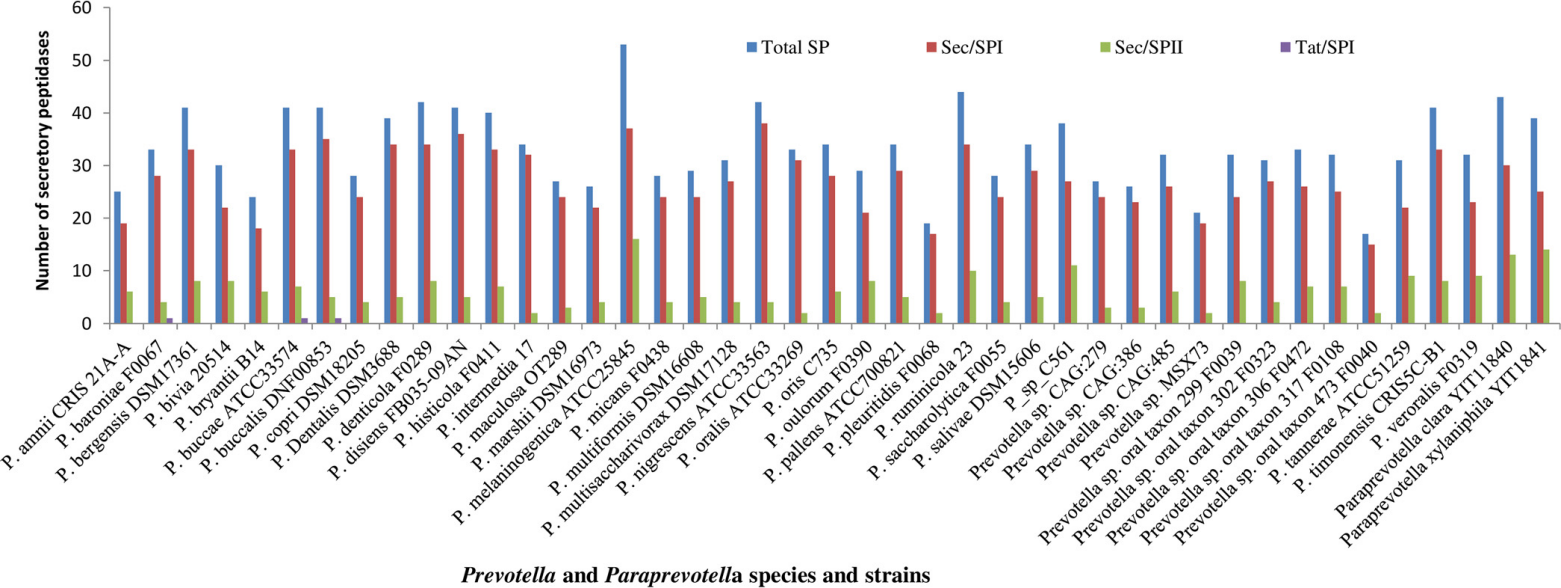
Secretory types of peptidases (peptidases with a signal peptide) found in the genomes of Prevotella and Paraprevotella species and strains. Total SP, total secretory peptidases; Sec/SPI, secretory peptidases with a signal peptide transported by secretion translocation and cleaved by signal peptidase I; Sec/SPII, secretory peptidases with a lipoprotein signal peptide transported by secretion translocation and cleaved by signal peptidase II; Tat/SPI, secretory peptidases with a Tat signal peptide transported by twin-arginine translocation and cleaved by signal peptidase I.

Of all the secretory peptidases, Sec/SPI was more prevalent in all species/strains than other secretory types, which was more predominant in Prevotella nigrescens (*n* = 38), P. melaninogenica (*n* = 37), and P. disiens (*n* = 36) than in other Prevotella and Paraprevotella species/strains (Fig. 3). Among the species/strains of Prevotella and Paraprevotella, P. melaninogenica had the most secretory peptidases (*n* = 53), followed by P. ruminicola (*n* = 44), Paraprevotella clara (*n* = 43), P. denticola (*n* = 42), and P. nigrescens (*n* = 42), while Prevotella sp. oral taxon 473 F0040 (*n* = 17) and Prevotella pleuritidis (*n* = 19) had the fewest secretory peptidases. Metallo, serine, and cysteine peptidases had more secretory peptidases than the other catalytic types (Fig. 2). Sec/SPII was more predominant in P. melaninogenica (*n* = 16), Paraprevotella xylaniphila (*n* = 14), and P. clara (*n* = 13) than in other Prevotella and Paraprevotella species/strains.

### Comparison of the species/strains for their peptidase occurrence patterns.

**FIG 4:**
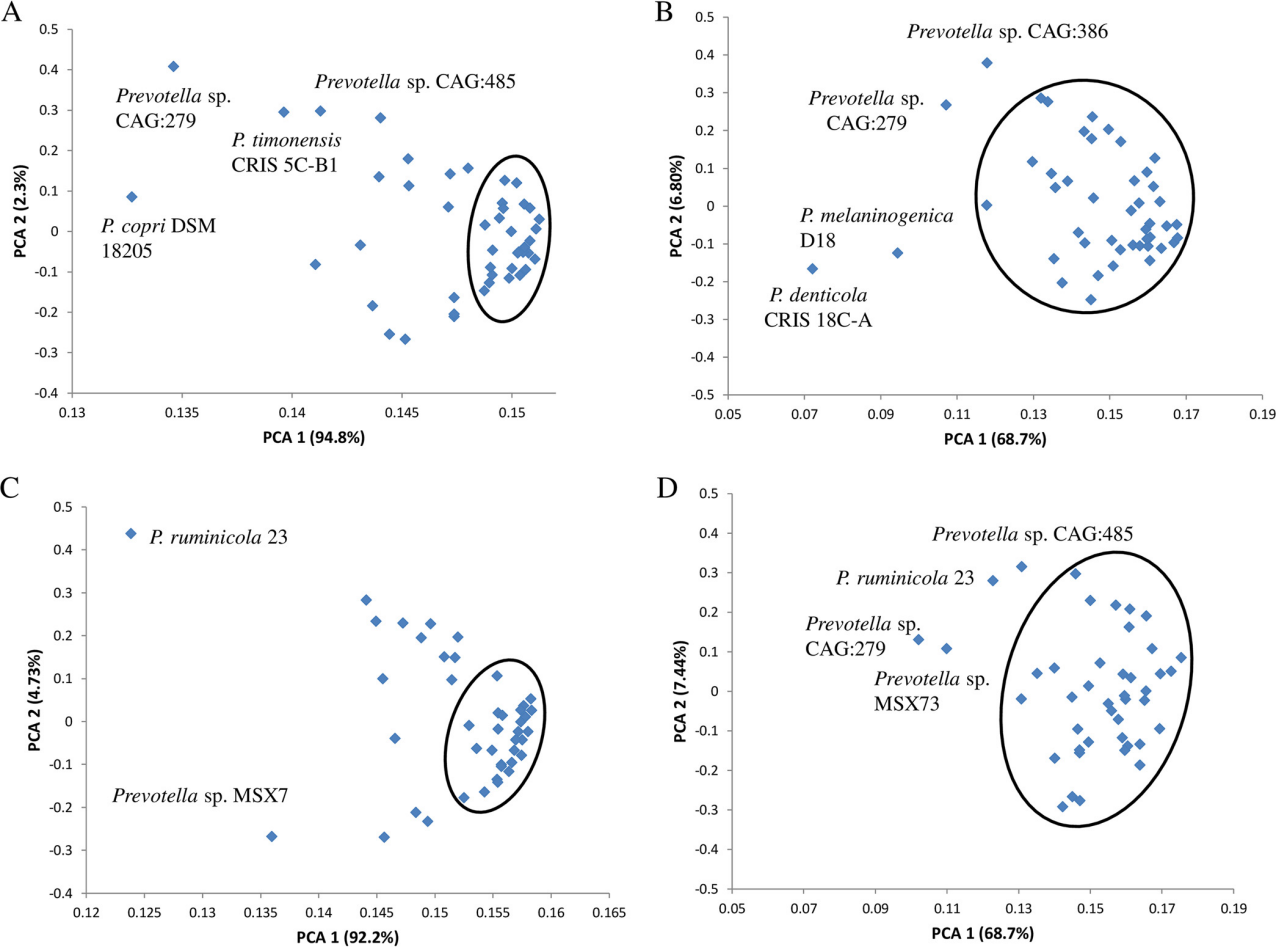
Principal-component analysis (PCA) of peptidase occurrence based on the distribution of catalytic types of all the peptidases (A) and secretory peptidases (C) and the distribution of peptidase families of all peptidases (B) and secretory peptidases (D).

Principal-component analysis based on total peptidases and their occurrence at the catalytic type and family levels showed that PCA1 accounted for most of the variations (94.8 and 68.7% at the catalytic type and family levels, respectively) (Fig. 4A). Based on the occurrence of catalytic types, more than 50% of the species formed a tight cluster, and the remaining species scattered along both the PC1 and PC2 axes, with Prevotella copri DSM 18205, Prevotella sp. CAG:279, P. timonensis CRIS 5C-B1, and Prevotella sp. CAG:485 scattered further away. However, all but P. denticola CRIS 18C-A, P. melaninogenica D18, Prevotella sp. CAG:279, and Prevotella sp. CAG:386 formed one cluster when analyzed based on the occurrence of peptidase families (Fig. 4B).

For secretory peptidases, PCA1 also accounted for most of the variation (94.8 and 68.7% at the catalytic type and family levels, respectively) (Fig. 4C). Similar to all the peptidases, most species formed one tight cluster, and the remaining species scattered along both the PC1 and PC2 axes, with P. ruminicola 23 and Prevotella sp. MSX73 scattering further away. At the family level of secretory peptidases, all but P. ruminicola 23, Prevotella sp. CAG:279, Prevotella sp. CAG:485, and Prevotella sp. MSX73 formed one large cluster (Fig. 4D).

**FIG 5:**
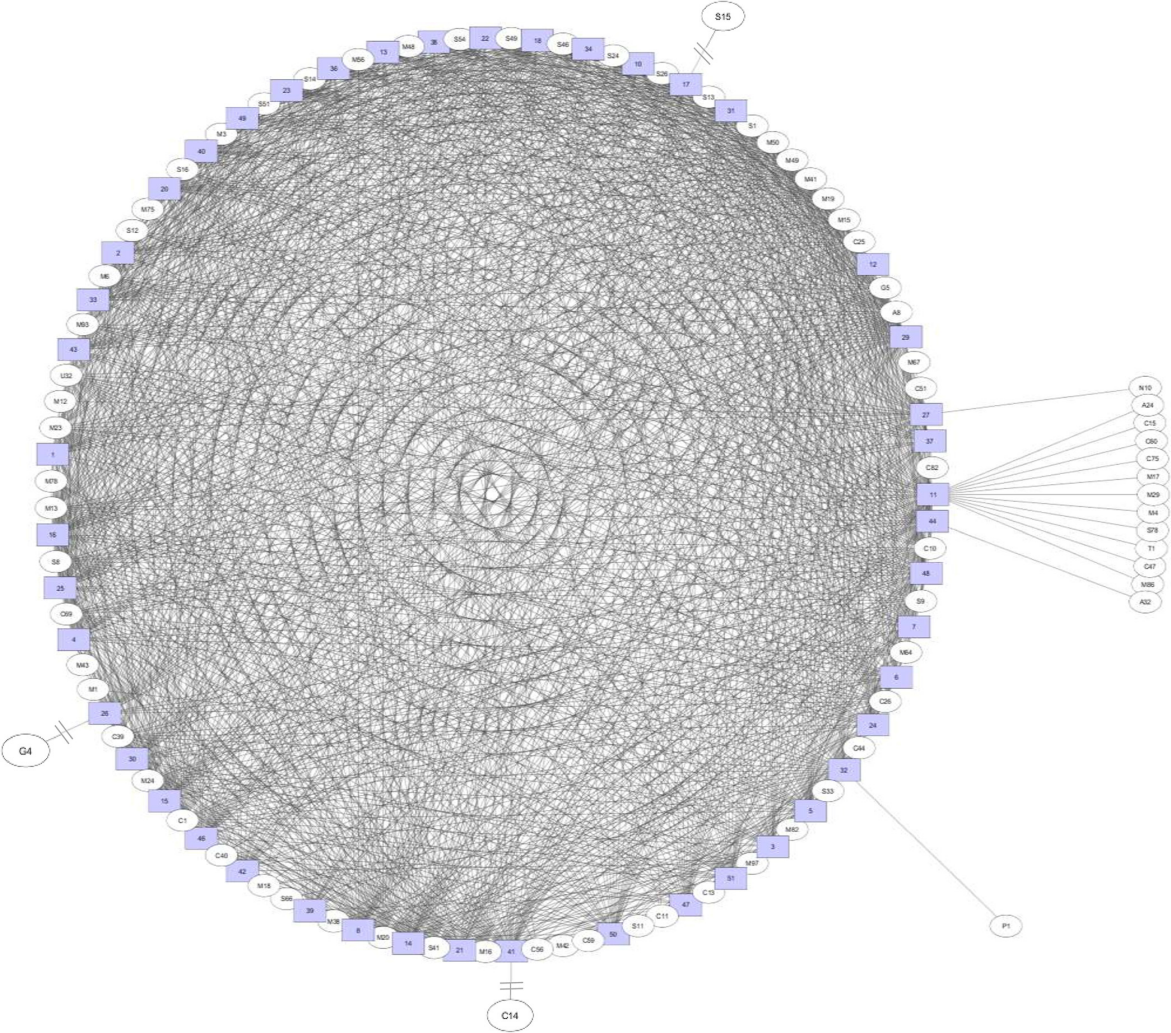
A bipartite association network comparing the occurrence of peptidase families. The peptidase families are shown as ovals, while bacterial species names are shown as numbers in square boxes. 1, P. amnii CRIS 21A-A; 2, P. baroniae F0067; 3, P. bergensis DSM 17361; 4, P. bivia DSM 20514; 5, P. bryantii B14; 6, P. buccae ATCC 33574; 7, P. buccae D17; 8, P. buccalis DNF00853; 10, P. copri DSM 18205; 11, P. dentalis DSM 3688; 12, P. denticola CRIS 18C-A; 13, P. denticola F0289; 14, P. disiens FB035-09AN; 15, P. histicola F0411; 16, P. intermedia 17; 17, P. maculosa OT289; 18, P. marshii DSM 16973; 20, P. melaninogenica ATCC 25845; 21, P. melaninogenica D18; 22, P. micans F0438; 23, P. multiformis DSM 16608; 24, P. multisaccharivorax DSM 17128; 25, P. nigrescens ATCC 33563; 26, P. oralis ATCC 33269; 27, P. oris C735; 29, P. oulorum F0390; 30, P. pallens ATCC 700821; 31, P. pleuritidis F0068; 32, P. ruminicola 23; 33, P. saccharolytica F0055; 34, P. salivae DSM 15606; 36, Prevotella sp. C561; 37, Prevotella sp. CAG:279; 38, Prevotella sp. CAG:386; 39, Prevotella sp. CAG:485; 40, Prevotella sp. MSX73; 41, Prevotella sp. oral taxon 299 F0039; 42, Prevotella sp. oral taxon 302 F0323; 43, Prevotella sp. oral taxon 306 F0472; 44, Prevotella sp. oral taxon 317 F0108; 46, Prevotella sp. oral taxon 473 F004; 47, P. tannerae ATCC 51259; 48, P. timonensis CRIS 5C-B1; 49, P. veroralis F0319; 50, P. clara YIT 11840; 51, P. xylaniphila YIT 11841.

The bipartite association network for total peptidase families revealed that most of the peptidase families were shared among Prevotella species (Fig. 5). Only 16 peptidase families were unique to some Prevotella species, with S15 for Prevotella maculosa OT 289, G4 for Prevotella oralis ATCC 33269, P1 (mixed) for P. ruminicola 23, A32 for Prevotella sp. oral taxon 317 F0108, and N10 for Prevotella oris C735 (Fig. 5). P. dentalis DSM 3688 had many unique peptidase families (i.e., A24, C15, C47, C60, C75, M4, M17, M29, M86, S78, and T1) that were not present in the other Prevotella species (Fig. 5).

**FIG 6:**
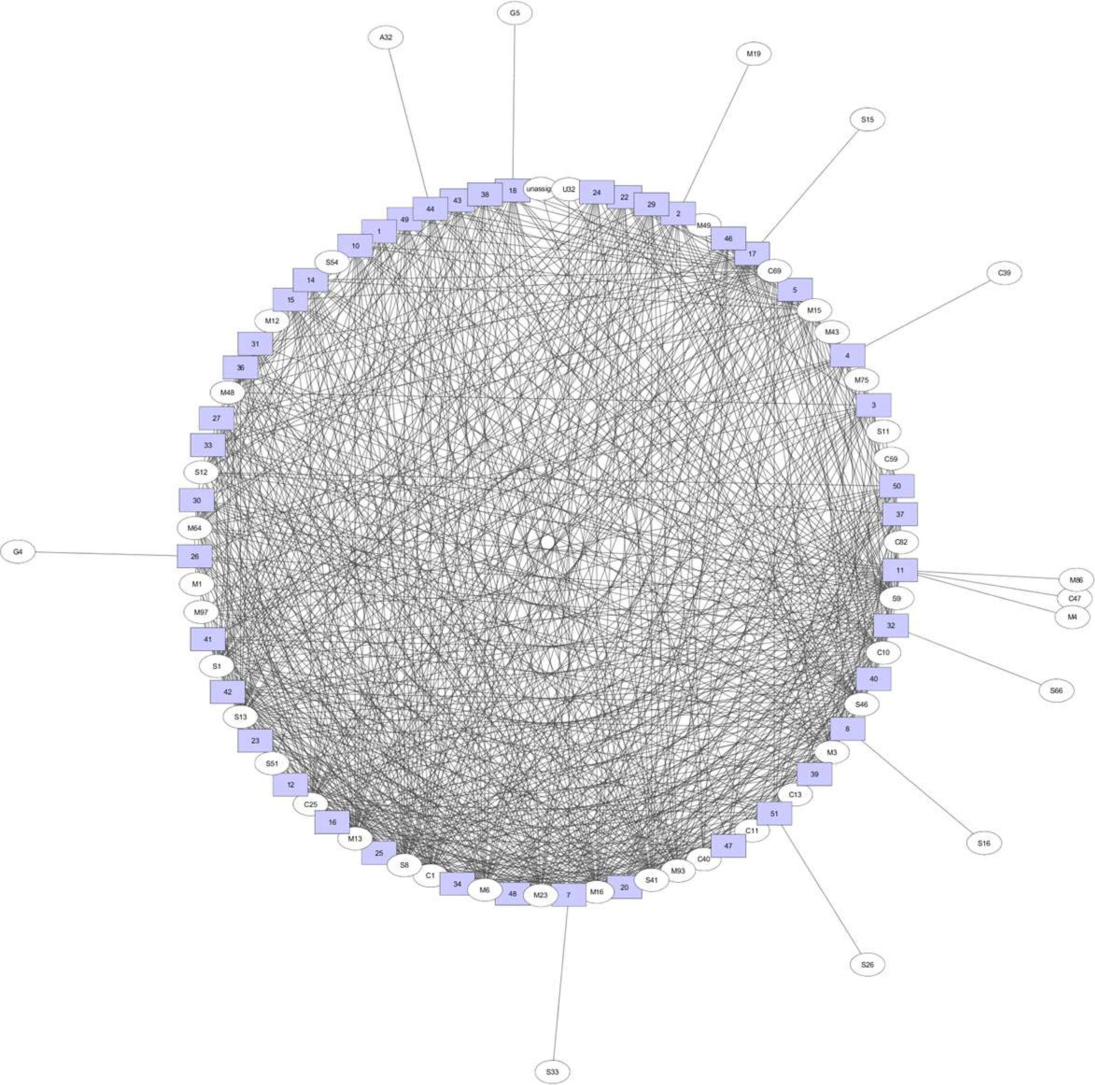
A bipartite association network comparing the occurrence of secretory peptidase families. The peptidase families are shown as ovals, while bacterial species names are shown as numbers in square boxes. See Fig. 5 for the species names corresponding to the numbers.

**FIG 7:**
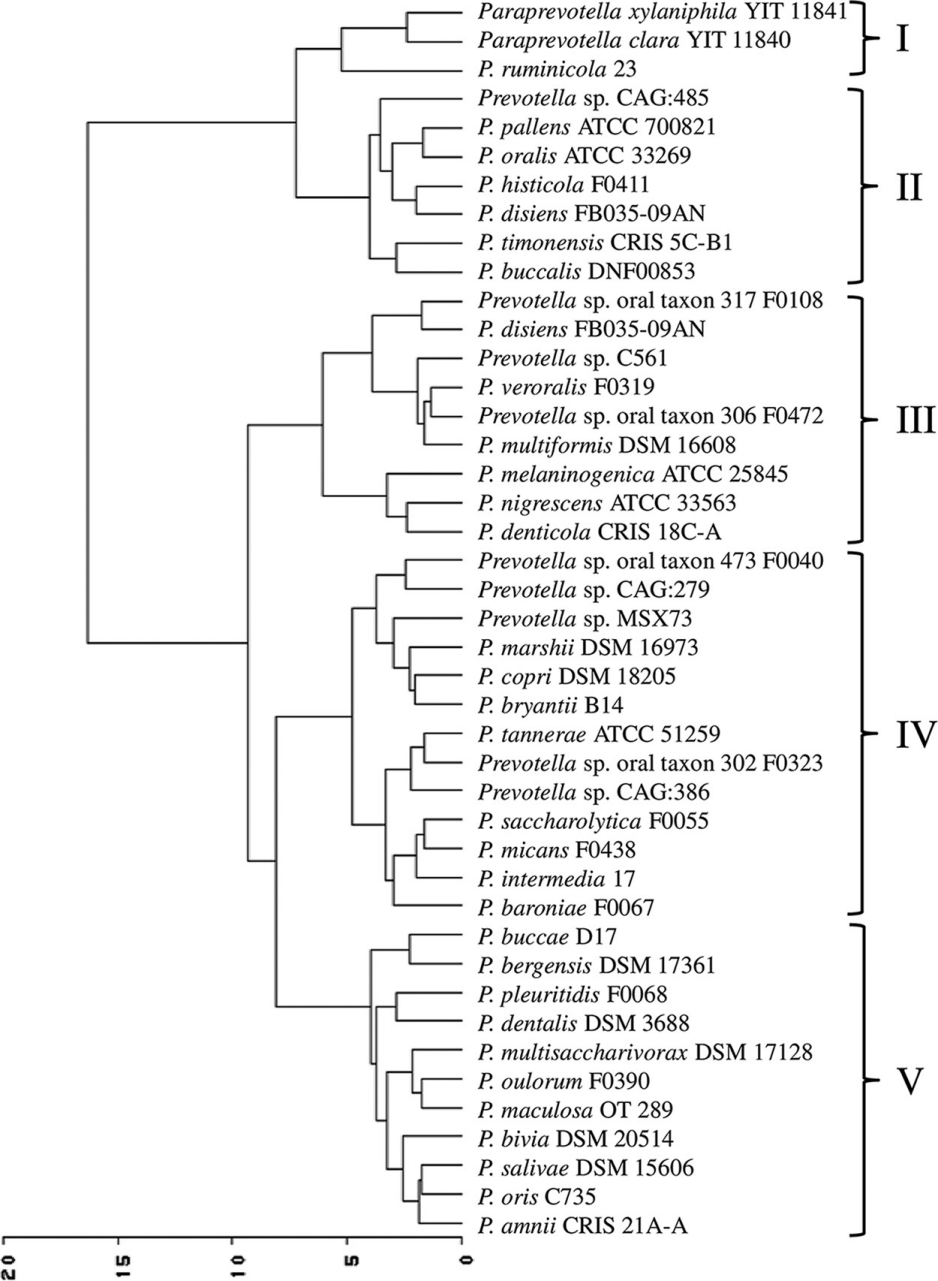
Ward cluster analysis of secretory peptidase families in different species of Prevotella and Paraprevotella.

For secretory peptidases, 13 peptidase families were exclusively found in some Prevotella species: G4 in P. oralis ATCC 33269, G5 in Prevotella marshii DSM 16973, M19 in Prevotella baroniae F0067, S15 in P. maculosa OT 289, S66 in P. ruminicola 23, S16 in P. buccalis DNF00853, S26 in P. xylaniphila YIT 11841, S33 in P. buccae D17, C39 in *P. bivia* DSM 20514, and A32 in Prevotella sp. oral taxon 317 F0108 (Fig. 6). Same as for total peptidases, P. dentalis DSM 3688 had three unique secretory peptidase families (i.e., M4, M86, and C47 families) that were not present in the other Prevotella species (Fig. 6). Cluster analysis of the secretory peptidase families revealed five clusters, with P. ruminicola and the two Paraprevotella species (P. clara and P. xylaniphila) forming one cluster and Prevotella sp. CAG:485, P. pallens, P. oralis, P. histicola, P. disiens, P. timonensis, and P. buccalis forming another cluster, and both clusters were separated from the clusters containing the other species (Fig. 7).

**FIG 8:**
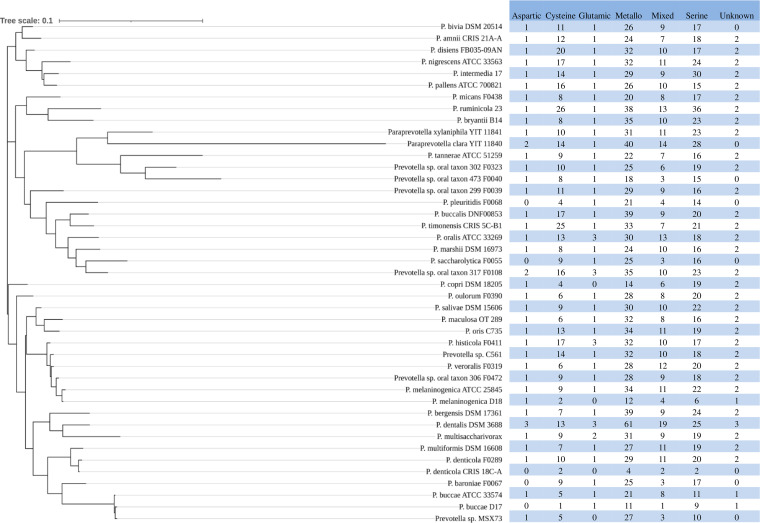
Phylogenetic tree of the Prevotella and Paraprevotella species showing the distribution of different catalytic types of peptidases.

The phylogenetic tree constructed based on the 16S rRNA gene sequences of the Prevotella and Paraprevotella species along with the distribution of catalytic types of all the peptidases are presented in Fig. 8. For most species, their phylogenetic relationship did not correspond to their relationship that was based on the occurrence of peptidases. The two strains of the P. melaninogenica differed in the occurrence of all except aspartic peptidase. The two strains of P. buccae also differed in their occurrence of most catalytic types of peptidases.

**FIG 9:**
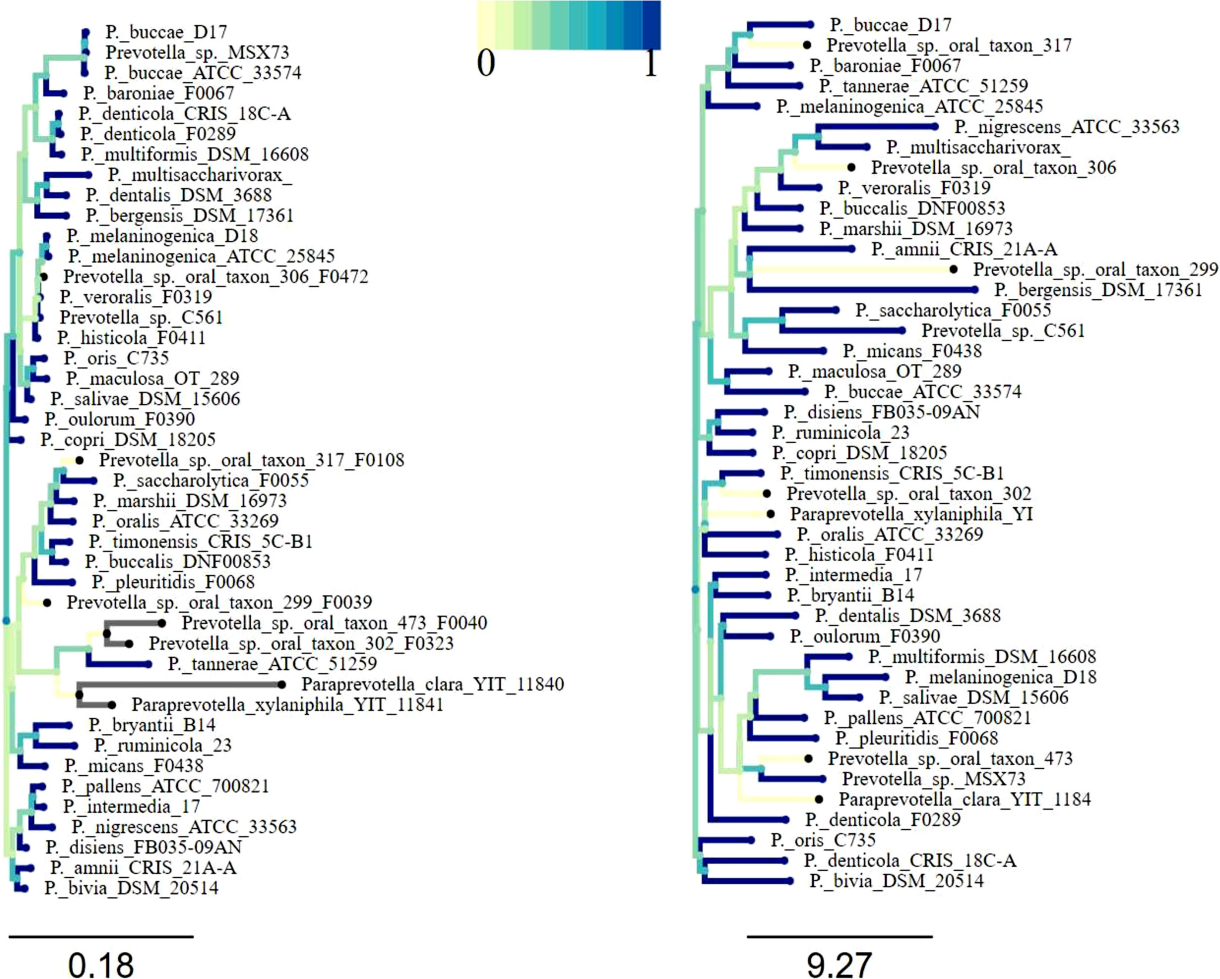
A phylogenetic tree based on 16S rRNA gene sequences (left) and a cladogram based on a distance matrix of the distribution of peptidase families (right). The deviation/distance from the blue to the light green lines corresponds to the dissimilarity at the branches of the phylogenetic tree and cladogram.

The phylogenetic tree based on 16S rRNA gene sequences and the cladogram based on the distribution matrix of peptidase families shared no similarity in topology (Fig. 9). The distribution of all the peptidases at the family level was not found significantly correlated with the 16S rRNA gene sequence-based phylogenetic relationships among the species (rMantel = −0.041 to −0.044, simulated *P* value = 0.55 to 0.59, based on more than 1,000 replicates) (Fig. 9, right panel). The correlation between the distribution distance matrix of secretory peptidase families and the 16S rRNA phylogenetic distance matrix was also not significant (rMantel = −0.051, simulated *P* value = 0.61 to 0.63, based on more than 1,000 replicates) (correlation tree not shown).

## DISCUSSION

The MEROPS database classifies the peptidases into eight distinct catalytic types, and Prevotella and Paraprevotella genomes encode six of them (i.e., asparagine and threonine peptidases were absent). One peptidase analysis of 128 genomes of *Bacteria*, *Archaea*, and *Eukarya* revealed that serine (41%), metallo (29%), and cysteine (22%) peptidases were most prevalent (37). In the study presented here, metallo (40.4%), serine (26.9%), and cysteine (15.4%) peptidases were most prevalent, indicating that Prevotella and Paraprevotella species have different peptidase occurrence patterns compared to the global peptidase patterns. In three of the Prevotella species (i.e., P. copri DSM 18205, P. intermedia 17, and Prevotella sp. CAG:279), serine peptidases were, however, more abundant than metallopeptidases. At the family level, the families S9 (prolyl oligopeptidase), M20 (glutamate carboxypeptidase), M16 (pitrilysin), C10 (streptopain), and C26 (γ-glutamyl hydrolase) were most abundant, and the families M16, S41 (C-terminal processing peptidase-1), and C1 (papain) were ubiquitous. Most members of M16 and S41 are involved in protein processing (38, 39). Various Prevotella species such as P. oralis, P. melaninogenica, P. intermedia, P. nigrescens, P. buccae, and P. denticola are implicated in dentoalveolar (purulent) infection (40). The peptidases of these Prevotella species may be involved in the degradation of connective tissue in gingiva, breakdown of proteinaceous compounds involved in the defense mechanisms in hosts, and virulence in the pathogenicity of oral infections (40, 41). In one study with different Prevotella species (one strain for each species), P. intermedia and P. nigrescens were shown to have much higher peptidase activities than P. buccae, P. denticola, P. melaninogenica, and P. oralis (21.1 to 23.5 versus 8.5 to 10.5 × 10^−8^ A-units) (40). In the present study, we found that the two “high-protease-activity” Prevotella species (P. intermedia and P. nigrescens) contained more peptidases (86 and 88 peptidases, respectively) than the four “low-protease-activity” species (P. buccae, P. denticola, P. melaninogenica, and P. oralis; 36, 42, 53, and 80 peptidases, respectively), and this suggests that Prevotella species that carry a large number of peptidases may be more proteolytic.

In the large intestines of humans and animals and the rumen of ruminants, protein degradation is primarily driven by proteolytic bacteria, including species of Prevotella. In the rumen, excessive proteolysis leads to decreased dietary nitrogen utilization efficiency (42). Inhibition of some of the secretory peptidases of Prevotella species may help reduce their proteolytic activities to achieve improved nitrogen utilization. Indeed, inhibitors of dipeptidyl peptidase decreased ammonia concentration in cultures of P. albensis, P. ruminicola, P. bryantii, and P. brevis and in mixed cultures of ruminal bacteria (43). The decreased ammonia concentration indicates reduced protein degradation and production of ammonia, which cannot be assimilated by ruminants for protein synthesis, and increased dipeptides, which can be utilized by ruminants. In the present study, S9 peptidases (prolyl oligopeptidase, dipeptidyl peptidase IV, and acylaminoacyl peptidase) were found, and they may be targets for inhibition to improve dietary nitrogen utilization efficiency in ruminants.

Most nascent proteins destined for secretion into the extracellular milieu contain a signal peptide at the N terminus (44). After signal peptides are cleaved off during the secretion process, the mature secretory protein is exported to extracellular environments through different secretion systems (44, 45). Secretory proteins can be predicted based on the presence of a signal peptide. We used SignalP to predict the signal peptides of the peptidases. In one study, SignalP and Phobius (another software tool to predict signal peptides) were shown to produce nearly identical predictions (32). It has been predicted that 7.8 to 10.5% of the proteins of various mycobacterial proteomes (46) and one-third of the entire eukaryotic proteome (45) are putative secretory proteins. Although total proteins in proteomes were not analyzed in the present study, secretory peptidases accounted for 45.5% of the total peptidases present in the Prevotella and Paraprevotella species. In *Bacteria* and *Archaea*, serine, metallo, and cysteine secretory peptidases represent more than 80% of the secretory peptidase with serine peptidases accounting for more than 50% (32). In the present study, we found that nearly 90% of the secretory peptidases of the Prevotella and Paraprevotella species, similar to total peptidases, were metallo (37.8%), serine (28.8%), or cysteine (23.3%) peptidases. Compared with the distributions of secretory peptidases (about 48% serine, 31% metallo, and 17% cysteine peptidases; estimated from Fig. 1 of reference [32]) in the *Bacteria* kingdom, the secretory peptidases among the Prevotella species were enriched with more metallopeptidase and cysteine peptidase at the expense of serine peptidase. Future research can help determine the actual contribution of these three types of secretory peptidases to the overall protein degradation in the rumen.

The abundance of secretory peptidases varies widely between the *Archaea* and *Bacteria* kingdoms (32), and the present study revealed extensive variations even among the species of Prevotella and Paraprevotella. Large variations in the genetic diversity and proteolytic activities among Prevotella species have been reported earlier (28). Differences in the number of peptidase families, including secretory peptidases, may reflect their niche adaptation, such as to temperature, pH, substrate, and competitors (19, 32, 47, 48). Among the secretory peptidases, standard secretory signal peptides transported by secretion translocation and cleaved by signal peptidase I (Sec/SPI) was predominant (81.3%), followed by Sec/SPII (18.4%) in the Prevotella species, and these patterns of Sec/SPI and Sec/SPII peptidases were noted in other organisms (45, 46).

Intracellular peptidases are usually associated with regulatory functions and protein turnover, whereas secretory or extracellular peptidases (both free and membrane-bound) are involved in the enzymatic degradation of proteinaceous materials for the acquisition of carbon and nitrogen from the environment (32, 49). As revealed in the present study, the number of secretory peptidases varied considerably among the species; P. melaninogenica ATCC 25845 had the greatest number of secretory peptidases, followed by P. ruminicola 23 and P. clara YIT 11840. Different from most bacterial species, P. melaninogenica ATCC 25845 possesses two chromosomes: the primary chromosome carrying 1,318 coding genes and the secondary chromosome carrying 978 coding genes (19). P. melaninogenica is an important human pathogen of the upper respiratory tract, and it is the most prevalent and abundant anaerobic bacterium in cystic fibrosis lungs (50). This species has the most secretory peptidases (53 in total and 36 Sec/SPI type), and these secretory peptidases may make it a prevalent and abundant pathogen. Secretory peptidases vary between *Bacteria* and *Archaea* and between Gram-positive and Gram-negative bacteria (32). A study on several rumen Prevotella species also found great variability in their proteolytic activities (28). The greater number of secretory peptidases may correlate to a broader range of proteolytic activity of the species.

It will be interesting to compare the repertoires of the peptidases and the actual proteolytic activities between Prevotella species, but few studies have compared the proteolytic activities of multiple Prevotella species. P. ruminicola 23 had more total secretory peptidases than P. bryantii B14 (44 versus 24). P. ruminicola had the greatest range and activity of dipeptidyl peptidases (30, 51), which release dipeptides from the N terminus of peptides. The families C40, M49, S9, S15, and S46 contain dipeptidyl peptidases. P. ruminicola 23 had more secretory dipeptidyl peptidases (one C40, eight S9, and one S46) than P. bryantii B14 (two S9 and one S46). The differences in the abundance of secretary peptidases corroborate the greater extracellular peptidase activities observed in P. ruminicola D31d than in P. bryantii B14 (28). P. ruminicola 23 also had more secretory cysteine and serine peptidases than P. bryantii B14 (20 versus 5 and 15 versus 8, respectively). These two types of secretory peptidases may play a greater role in proteolysis by P. ruminicola than by P. bryantii. Indeed, specific inhibitors of cysteine peptidase (iodoacetate) inhibited the extracellular peptidase activity of P. ruminicola to a greater extent than that of P. bryantii (28). Furthermore, P. ruminicola 23 and P. bryantii B14 had similar numbers of secretory metallopeptidases (11 versus 9), and specific inhibition of metallopeptidases by 1,10-phenanthroline decreased the extracellular peptidase activities of these two strains to similar degrees (28). The sequencing of the genome of P. ruminicola D31d has not been completed, and thus its repertoire of peptidases is not known and cannot be directly compared to that of P. ruminicola 23. However, the difference in the repertoire of secretory peptidases between P. ruminicola 23 and P. bryantii B14 parallels the differences in the extracellular peptidase activities and in their response to metallopeptidase inhibition between P. ruminicola D31d and P. bryantii B14. It is reasonable to postulate that the type and abundance of peptidases identified from the sequenced genomes of Prevotella and Paraprevotella species can serve as a proxy of their proteolytic capacity and activities. However, although P. ruminicola 23 had more secretory serine peptidase than P. bryantii B14, phenylmethylsulfonyl fluoride, a specific inhibitor of serine peptidases, inhibited the extracellular peptidase activity of the latter to a greater extent than that of the former (28). The reason is unclear, but differences between strains 23 and D31d (52) may also determine the differences in their proteolytic activities along with protein substrates. Once the sequencing of the genome of P. ruminicola D31d is completed, the repertoire of the secretary peptidases and their activities can be compared between P. ruminicola D31d and P. bryantii B14.

Bipartite network analysis revealed that most peptidase families were shared among the Prevotella and Paraprevotella species, but P. dentalis had a few peptidase families that were not rare in the other species. P. dentalis inhabits the periodontal area of oral cavities, and it is predominantly responsible for periodontal diseases (20). It is not clear whether these unique peptidases are the results of the adaptation to this habitat by P. dentalis. Principal-component analysis based on total peptidase families showed that most of the bacterial species formed one cluster except for a few exceptions. Especially, Prevotella sp. CAG:279, Prevotella sp. CAG:485, and Prevotella sp. CAG:386 were separated from the other species. The 16S rRNA gene sequences of these three strains are not available in Silva or other databases, so we could not determine their phylogenetic relatedness with the other species. Based on the secretory peptidases, most of the bacterial species also formed one cluster except P. ruminicola 23, Prevotella sp. MSX73, Prevotella sp. CAG:279, and Prevotella sp. CAG:485. Different strains of P. ruminicola can occupy different ecological niches, but P. ruminicola 23 is solely present in the rumen along with P. brevis GA33 (53, 54). Although P. bryantii B14 is frequently observed in the rumen, it is not closely related to P. ruminicola 23 as shown in the left panel of Fig. 9 (only 89.7% 16S rRNA gene sequence similarity). It has also been demonstrated from 16S rRNA analysis that P. bryantii B14 clustered with a nonruminal Prevotella supercluster that has additional ecological niches such as the oral cavity, whereas P. ruminicola 23 clustered with a ruminal Prevotella supercluster (54). Cluster analysis based on the secretory peptidase families indicated that P. ruminicola, P. clara, and P. xylaniphila formed a distinct cluster. Although frequently reported in the rumen (55–57), the role of the genus Paraprevotella therein is not well defined, P. clara and P. xylaniphila may be involved in proteolysis as the well-characterized ruminal P. ruminicola owing to their similar secretory peptidases.

The cladogram based on total peptidase families or the secretory peptidases and the phylogenetic tree based on the 16S rRNA gene sequences had different topologies across the Prevotella and Paraprevotella species. This contradicts the report that the distribution of secretory peptidases correlated significantly with the 16S rRNA-based phylogeny within the *Archaea* or *Bacteria* kingdom, and such correlation was most significant between closely related taxa for both *Archaea* and *Bacteria* though the correlations were weak (32). Among the species analyzed in the present study, the distribution of peptidases may not reflect their phylogenetic relationship, and recruitment of peptidases and speciation might not be closely evolutionally linked.

In conclusion, Prevotella and Paraprevotella species possess 6 distinct catalytic types and 78 families (48 families of secretory peptidases) of peptidases, with metallopeptidases being present in the highest proportion, followed by serine and cysteine peptidases. The number and families of peptidases varied considerably among the species, but a few families of peptidases were ubiquitous to all species. Based on the peptidase distribution patterns, the majority of the species clustered together with a few exceptions. The bipartite association network based on the peptidase families revealed that most of the peptidase families were shared among the species. This study did not reveal any significant correlation between the phylogenetic relationship and the distribution of peptidase families, suggesting no evolutionary linkage of peptidases among the species. The results of this study could be used to identify some secretory peptidases, especially those that are abundant and ubiquitous in the Prevotella species, which can be inhibited to reduce excessive dietary protein degradation in the rumen, improving protein utilization efficiency in ruminant animals.

## MATERIALS AND METHODS

### Collection of peptidase sequences and analyses.

Sequences and related data of the peptidases identified in the genomes of Prevotella and Paraprevotella species were downloaded from the MEROPS database (https://www.ebi.ac.uk/merops/) (35). In this database, peptidases are manually curated and hierarchically classified into eight catalytic types based on the catalytic residue serving at the active site of the enzymes, clans based on evolutionary origins, and families and then subfamilies based on AA sequence homology (35, 47, 58). When the database (release 12.3) was accessed (April 15, 2021), it had 16, 97, 6, 74, 10, 2, 54, 6, and 8 families (total 265) of peptidases for the aspartic, cysteine, glutamic, metallo, asparagine, mixed, serine, threonine, and unknown peptidases, respectively. Data relating to catalytic types, clan, family, MEROPS ID, or MERNUM (the MEROPS sequence identifier) of peptidases or nonpeptidase homologs (proteins of known sequences that can be placed in a peptidase family but lack one or more of the expected catalytic residues) of strains of Prevotella (*n* = 44) and Paraprevotella (*n* = 2), whose whole genomes had been completely sequenced, were extracted based on the Index of Organisms. The details of species and the information of the peptidases are shown in Table S5. The peptidase sequences of all the species of Prevotella (*n* = 41) and Paraprevotella (*n* = 2) were also downloaded. All the peptidases involved in the proteolytic degradation of dietary protein are extracellular enzymes, and they have a signal peptide (SP) to allow for their excretion. Therefore, we predicted the presence of SP (short AA sequences at the N terminus), with the “Gram-negative” option selected, among all the downloaded peptidase sequences (3,752 in total) using the SignalP-5.0 server (https://services.healthtech.dtu.dk/service.php?SignalP-5.0), which predicts the presence of SP and secretory types based on the deep neural network-based method (59). As a suitable method to predict prokaryotic secretory peptidases (32), the SignalP algorithm predicts proteins as standard secretory signal peptides transported by secretion translocation and cleaved by signal peptidase I (Sec/SPI), Tat signal peptides transported by twin-arginine translocation and cleaved by signal peptidase I (Tat/SPI), lipoprotein signal peptides transported by secretion translocation and cleaved by signal peptidase II (Sec/SPII), or no signal peptides (other). The prediction summary for each peptidase was downloaded from the SignalP-5.0 server. The MEROPS database does not have the AA sequences of peptidases for the individual strains of Prevotella and Paraprevotella. Therefore, species differences, but not strain variations, in the secretory peptidases of Prevotella and Paraprevotella species were extracted.

### Phylogenetic analysis and comparisons.

The taxonomy database identifiers of the individual species of Prevotella and Paraprevotella recorded in the MEROPS database were used to search for the corresponding 16S rRNA gene sequences in the SILVA database (release 138.1) using “NCBI tx ID” (60). The taxonomic identifiers present in both databases were used to identify the genomes of interest in this study. Only the completely annotated genomes with available 16S rRNA gene sequences in the SILVA database (release 138.1) were included in this study. The 16S rRNA gene sequences of Prevotella (*n* = 41) and Paraprevotella (*n* = 2) species were downloaded from the SILVA database as a Fasta file (60). The 16S rRNA gene sequences of Prevotella sp. CAG:279, Prevotella sp. CAG:386, and Prevotella sp. CAG:485 were not available in any databases. The 16S rRNA gene sequences were then aligned using the SILVA Incremental Aligner (SINA) (61), and the tree (as a Newick file) and FASTA files were downloaded. A phylogenetic distance matrix was obtained from the Newick file using the T-REX web-based server (62), which was then imported into R. A distance matrix of the bacterial species based on the distribution of peptidase (total and secretory peptidases) families (counts of individual families) was also constructed using the ade4 package in R (63). Then, a correlation between the phylogenetic distance matrix of 16S rRNA genes and the peptidase distribution distance matrix was evaluated using the Mantel test of ade4 in R (63). The phylogenetic distance matrix and the distance matrix of the peptidase family distribution in the Newick format were uploaded to the Plylo.io web server (http://phylo.io/) to visualize and compare them (64). The phylogenetic tree of Prevotella and Paraprevotella species and the distributions of different catalytic types of peptidases were visualized using the Interactive Tree Of Life (iTOL; version 6.1.1) online tool (65).

Principal-component analysis (PCA) was performed using “Proc Princomp” of SAS package (66) to explore the similarity of peptidase distributions among different Prevotella species and strains. The PCA was based on the correlation matrix of the numbers of peptidases, including secretory peptidases in various catalytic types and families in each Prevotella species.

The secretory peptidases were summarized in a matrix containing the counts of secretory peptidases assigned to peptidase families (rows) across all Prevotella species (columns). Indices between the secretory peptidases of individual species were calculated from this matrix using the Ward’s minimum-variance method and were used to generate a secretory peptidase distance matrix using the “Proc cluster” followed by the “Proc tree” procedures of the SAS package (66). A bipartite association network of unique and shared families of peptidases in Prevotella species was generated and visualized using Cytoscape release 3.6.1 (67).

### Data availability.

All the data are presented in the tables or in the supplementary files.
