# Supplementary material for: Genomic Insights into the Distribution of Peptidases and Proteolytic Capacity among Prevotella and Paraprevotella Species
Source: Microbiol Spectr. 2022 Apr 4;10(2):e02185-21. doi: 10.1128/spectrum.02185-21 (PMC9045265; doi:10.1128/spectrum.02185-21)
Supplement: SUPPLEMENTAL FILE 1 — Supplemental material. Download SPECTRUM02185-21_Supp_1_seq5.pdf, PDF file, 0.04 MB [file spectrum02185-21_supp_1_seq5.pdf]

SUPPLEMENTAL MATERIALS

Title of the supplementary tables and legend of the figure

Supplementary Table S1 Different families of peptidases found in *Prevotella* and *Paraprevotella* species/strains. The peptidase families (out of 78 in total) present in less than 50% of the bacterial species/strains are shown.

Supplementary Table S2 Occurrence of different families of peptidases among the species/strains of *Prevotella* and *Paraprevotella*.

Supplementary Table S3 Different families of secretory peptidases found in *Prevotella* and *Paraprevotella* species. The secretory peptidase families (out of 48 in total) present in less than 50% of the bacterial species are shown.

Supplementary Table S4 Occurrence of different families of secretory peptidases among the species/strains of *Prevotella* and *Paraprevotella*.

Supplementary Table S5 The species and strains of *Prevotella* and *Paraprevotella* and their peptidases analyzed.

Supplementary Fig. S1 The number (in brackets) and percentage of each catalytic type of peptidases found in the genomes of *Prevotella* and *Paraprevotella* species.

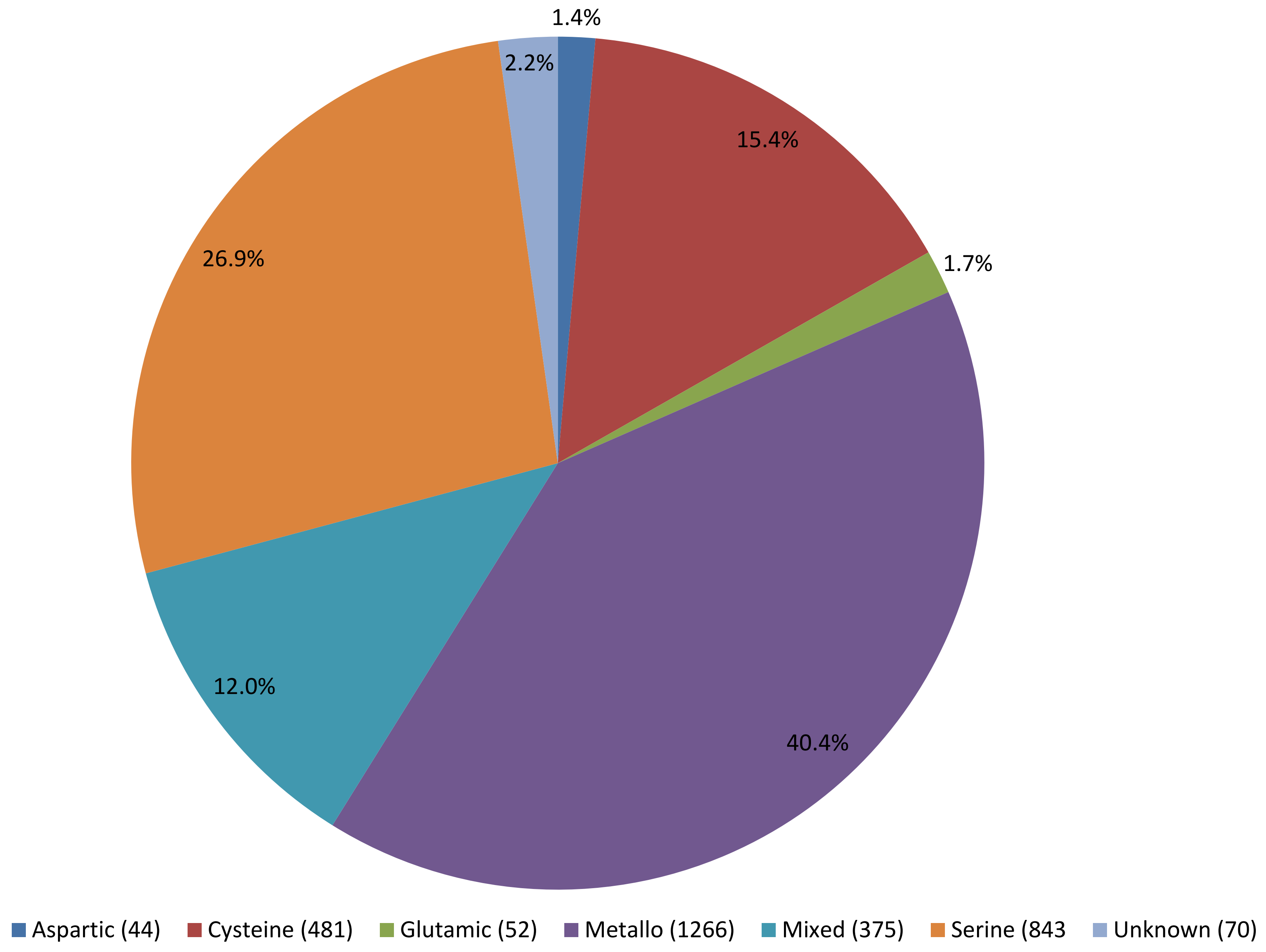

Supplementary Fig S1 The number (in brackets) and percentage of each catalytic type of peptidases found in the genomes of *Prevotella* and *Paraprevotella* species.

Supplementary Table S1 Different families of peptidases found in *Prevotella* and *Paraprevotella* species/strains. The peptidase families (out of 78 in total) present in less than 50% of the bacterial species/strains are shown here.

Peptidase family	Total number	% of total peptidases	Total carrying species/strains	% of total carrying species/strain
M56	31	0.99	22	47.8
M64	22	0.70	22	47.8
S12	23	0.73	18	39.1
S24	24	0.77	17	37.0
S51	19	0.61	16	34.8
C40	18	0.57	13	28.3
M97	16	0.51	13	28.3
C11	11	0.35	9	19.6
C13	12	0.38	8	17.4
C44	9	0.29	8	17.4
M18	8	0.26	7	15.2
M38	6	0.19	6	13.0
C56	5	0.16	5	10.9
M1	5	0.16	5	10.9
M75	5	0.16	5	10.9
M78	5	0.16	5	10.9
M82	5	0.16	5	10.9
C59	4	0.13	4	8.70
C51	3	0.10	3	6.52
S11	3	0.10	3	6.52
S33	3	0.10	3	6.52
S66	3	0.10	3	6.52
M42	4	0.13	2	4.35
C82	2	0.06	2	4.35
M12	2	0.06	2	4.35
M43	2	0.06	2	4.35
M67	2	0.06	2	4.35
M86	3	0.10	1	2.17
C47	2	0.06	1	2.17
A24	1	0.03	1	2.17
A32	1	0.03	1	2.17
C14	1	0.03	1	2.17
C15	1	0.03	1	2.17
C60	1	0.03	1	2.17

C75	1	0.03	1	2.17
G4	1	0.03	1	2.17
M17	1	0.03	1	2.17
M29	1	0.03	1	2.17
M4	1	0.03	1	2.17
N10	1	0.03	1	2.17
P1	1	0.03	1	2.17
S15	1	0.03	1	2.17
S78	1	0.03	1	2.17
T1	1	0.03	1	2.17

Supplementary Table S3 Different families of secretory peptidases found in *Prevotella* and *Paraprevotella* species. The secretory peptidase families (out of 48 in total) present in less than 50% of the bacterial species are shown here.

Peptidase family	Total number	% of total secretory peptidases	Total carrying species	% of total carrying species/strains
M64	20	1.40	20	46.5
S12	20	1.40	17	39.5
M97	17	1.19	13	30.2
C40	16	1.12	12	27.9
C13	13	0.91	9	20.9
C11	8	0.56	7	16.3
M1	5	0.35	5	11.6
M75	5	0.35	5	11.6
M49	4	0.28	4	9.3
S51	4	0.28	4	9.3
Unassigned	4	0.28	4	9.3
U32	3	0.21	3	7.0
C59	2	0.14	2	4.7
C82	2	0.14	2	4.7
M12	2	0.14	2	4.7
M43	2	0.14	2	4.7
S11	2	0.14	2	4.7
S54	2	0.14	2	4.7
C47	2	0.14	1	2.3
A32	1	0.07	1	2.3
C39	1	0.07	1	2.3
G4	1	0.07	1	2.3
G5	1	0.07	1	2.3
M19	1	0.07	1	2.3
M4	1	0.07	1	2.3
M86	1	0.07	1	2.3
S15	1	0.07	1	2.3
S16	1	0.07	1	2.3
S26	1	0.07	1	2.3
S33	1	0.07	1	2.3
S66	1	0.07	1	2.3
